# Phytochemicals and biological studies of plants in genus *Hedysarum*

**DOI:** 10.1186/1752-153X-7-124

**Published:** 2013-07-18

**Authors:** Yinmao Dong, Dongyan Tang, Na Zhang, Yue Li, Chunhong Zhang, Li Li, Minhui Li

**Affiliations:** 1 Department of chemistry, Harbin Institute of Technology, Harbin 150000, China; 2 Baotou Medical College, No. 31 Construction Road, Baotou, Inner Mongolia 014060, P. R. China; 3 Beijing Key Lab of Plant Resources Research and Development, Beijing Technology and Business University, Beijing 100048, China

**Keywords:** *Hedysarum*, Chemical constituents, Pharmacology, Utilization, TCM

## Abstract

In China, several species (*Hedysarum polybotrys* Hand.-Mazz., *Hedysarum limprichtii* Hlbr., *Hedysarum vicioider* Turcz. *var. Taipeicum* Hand.-Mazz. Liu, *Hedysarum smithianum*, *et al*.) of genus *Hedysarum* have a long history of use in traditional Chinese medicine (TCM). In TCM, these plants are used to increase the energy of the body. To date, 155 compounds, including flavonoids, triterpenes, coumarins, lignanoids, nitrogen compounds, sterols, carbohydrates, fatty compounds, and benzofuran, have been isolated from plants of the genus *Hedysarum*. Various chemical constituents contribute to the antioxidant, anti-tumor, anti-aging, anti-diabetic, and anti-hypertensive properties of these plants. *Hedysarum* species are used to treat infestation with gastrointestinal nematodes and may support the immune system and peripheral nervous system. In the present review, we summarize the research on the phytochemistry and pharmacology of *Hedysarum* species, which will be useful for better utilization of these important species in TCM.

## Review

### Introduction

*Hedysarum* is a genus of the family Fabaceae that consists of about 300 species of annual and perennial herbs. *Hedysarum* species are widely distributed in the temperate northern hemisphere, including Asia, Europe, North Africa, and North America. In China, 42 *Hedysarum* species are native and their center of distribution is in northwestern and southwestern China, most notably the Hengduan Mountain region [1]. Traditional Chinese medicine (TCM) has been used in China for hundreds of years and continues to play an important role in clinical application. Many species of genus *Hedysarum*, such as *Hedysarum polybotrys* Hand.-Mazz., *Hedysarum limprichtii* Hlbr., *Hedysarum vicioider* Turcz. var. Taipeicum Hand.-Mazz. Liu, and *Hedysarum smithianum* have been employed in TCM to strengthen the immune system and improve the energy of the body. The root of *H. polybotrys*, which is commonly known as “Hongqi” or “Radix Hedysari” is an important component of various TCM formulas. *H. polybotrys* was recorded in Chinese Pharmacopoeia 2010 [2]. The traditional curative functions of Hongqi are to invigorate “Qi”, increase urination, and promote tissue regeneration [2]. Biological studies have shown that Hongqi, which contains a large quantity of polysaccharides, has anti-aging, antioxidant, anti-tumor, and anti-diabetic activity. Hongqi is reported to have great potential for use in modern health food and plant cosmetics [1-5]. The phytochemistry and pharmacology of *Hedysarum* species have attracted increasing worldwide attention among those involved in the research and development of new drugs. In the present review, we summarize and list all of the secondary metabolites that have been identified in plants of genus *Hedysarum* over the past few decades. We also consider the pharmacological activity of *Hedysarum* species, which will be useful for better utilization of the important *Hedysarum* species in TCM formulas.

### Chemical constituents

Over the past few decades, 155 chemical constituents have been isolated from plants of genus *Hedysarum* through different chromatography methods and were identified by the spectrum of ^1^H-NMR, ^13^C-NMR, 2D-NMR, HR/MS, et al. [3-5]. The chemical structures of these constituents include flavonoids (**1–79**), triterpenes and triterpenoid saponins (**80–91**), coumarins (**92–103**), lignanoids (**104–105**), nitrogen compounds (**106–112**), sterols (**113–117**), carbohydrates (**118–123**), fatty compounds (**124–135**), benzofuran (**136–145**), and others (**147–155**). The names and corresponding plant resources are compiled in Table 1 and the chemical structures are described in Additional file 1. In recent years, some polysaccharides were isolated from the species of genus *Hedysarum* by aqueous extract followed by precipitation with ethanol. The homogeneous polysaccharide was obtained after treated with Savage method and H_2_O_2_, and purified with and Sephadex G-200 gel filtration chromatography. Then GC, HPGC, GPC-MALLS, elemental analyzer, phenol sulfuric acid method and Bradford method were used to study the physicochemical property of the polysaccharides [6].

**Table 1:** **155 compounds of the genus *Hedysarum***

**No.**	**Compound class and name**	**Source ref.**	
*Flavones*			
**1**	4′-methoxy-7-hydroxyflavone	*H. polybotrys*	[4]
**2**	Narcissin	*H. multijugum*	[7]
**3**	Apigenin	*H. multijugum*	[7]
**4**	5,7-dihydroxy-4′-methoxyflavone	*H. scoparium*	[8]
**5**	Isorhamnetin	*H. setigerum*	[9]
**6**	3-O-Methylkaempferol	*H. setigerum*	[10]
**7**	Quercetin 3-*α*-L-rhamnofuranoside	*H. gmelinii*	[11]
		*H. neglectum*	[12]
**8**	Quercetin 3-*α*-L-arabinofuranoside	*H. gmelinii*	[11]
		*H. connatum*	[13]
		*H. alpinum*	[13]
**9**	Polystachoside (quercetin3-*β*-L-arabinofuranoside)	*H. komarovii*	[14]
		*H. sachalinense*	[15]
		*H. neglectum*	[12]
		*H. connatum*	[13]
		*H. alpinum*	[13]
**10**	Rhoifolin	*H. setigerum*	[9]
**11**	Linarin	*H. setigerum*	[9]
**12**	Diosmin	*H. setigerum*	[9]
**13**	Kaempferol-3-O-*α*-L-arabinofuranoside	*H. setigerum*	[10]
**14**	Rutin	*H. setigerum*	[10]
**15**	Neobudofficide	*H. setigerum*	[10]
**16**	Quercetin-3-O-*β*-glucopyranoside-7- O-*α*-rhamnofuranoside	*H. aericeum*	[16]
		*H. caucasium*	
**17**	Quercetin	*H. austrosibiricum*	[17]
		*H. aericeum*	[16]
		*H. caucasium*	[16]
		*H. setigerum*	[10]
		*H. connatum*	[13]
		*H. alpinum*	[13]
**18**	Kaempferol	*H. aericeum*	[16]
		*H. caucasium*	[16]
**19**	Isoquercitrin	*H. aericeum*	[16]
		*H. caucasium*	[16]
**20**	Hyperoside	*H. aericeum*	[16]
		*H. caucasium*	[16]
		*H. komarovii*	[14]
		*H. sachalinense*	[15]
		*H. neglectum*	[12]
		*H. connatum*	[13]
		*H. alpinum*	[13]
**21**	Quercitrin	*H. setigerum*	[10]
		*H. connatum*	[13]
		*H. alpinum*	[13]
*Flavonones*			
**22**	Naringenin-5,7-di-O-*β*-D-glucopyranoside	*H. multijugum*	[7]
**23**	Naringin	*H. polybotrys*	[18,19]
**24**	Naringenin	*H. polybotrys*	[20]
*Isoflavones*			
**25**	6″-O-acetylononin	*H. theinum*	[3]
**26**	Ononin-6″-O-malonate	*H. polybotrys*	[19,20]
**27**	Ononin	*H. polybotrys*	[18,19]
		*H. polybctrys*	[20,21]
		*H. multijugum*	[7]
		*H. kirghisorum*	[22,23]
		*H. semenovii*	[24]
		*H. austrosibiricum*	[25]
**28**	Formononetin	*H. theinum*	[3,26]
		*H. polybotrys* *H. polybotrys*	[19,20] [21,27]
		*H. multijugum*	[28]
		*H. semenovii*	[24]
		*H. austrosibiricum*	[25]
		*H. kirghisorum*	[22,23]
**29**	Calycosin	*H. polybctrys*	[21]
		*H. semenovii*	[24]
**30**	5,7-dihydroxy-6-C-prenyl-4′-methoxy -isoflavone (Gancaonin A)	*H. multijugum*	[29,30]
**31**	5,7-dihydroxy-6,8-di-C-prenyl-4′- methoxy-isoflavone	*H. multijugum*	[29,30]
		*H. scoparium*	[8]
**32**	5,7-dihydroxy-4′-methoxy-isoflavone	*H. multijugum*	[28]
		*H. kirghisorum*	[23]
**33**	5,7-dihydroxy-8-C-prenyl-4′-methoxy isoflavone (gancaonin M)	*H. multijugum*	[28,30]
**34**	Osajin 4′-methyl ether	*H. scoparium*	[8]
**35**	Sissotrin	*H. multijugum*	[7,30]
		*H. kirghisorum*	[22,23]
**36**	Afromosin-7-O-*β*-D-glucopyranoside	*H. semenovii*	[24]
**37**	Calycosin-7-O-*β*-D-glucopyranoside	*H. semenovii*	[24]
**38**	Warangalone 4′-methyl ether	*H. multijugum*	[7]
		*H. scoparium*	[8]
**39**	Afrormosin	*H. multijugum*	[7]
		*H. kirghisorum*	[22,23]
		*H. semenovii*	[24]
**40**	5-hydroxy-4′-methoxy-6-prenyl-2‴- hydroxyisopropylfurano [4,5:8,7]-isoflavone	*H. scoparium*	[8]
**41**	5-hydroxy-4′-methoxy-6-prenylfurano [4,5:6,7]-isoflavone	*H. scoparium*	[8]
**42**	5-hydroxy-4′-methoxy-8-prenylfurano [4,5:6,7]-isoflavone	*H. scoparium*	[8]
**43**	5-hydroxy-4′-methoxy-8-prenyl-2″ - hydroxyisopropyldihydrofurano [4,5:6,7]-isoflavone	*H. scoparium*	[8,31]
**44**	5-hydroxy-4′-methoxy-6-prenyl-2‴ - hydroxyisopropyldihydrofurano [4,5:8,7]-isoflavone	*H. scoparium*	[8,31]
**45**	5-hydroxy-4′-methoxy-8-prenyl-1″, 2″-peroxyl-3″,3″-dimethyidihydro pyrano [5,6:6,7]-isoflavone	*H. scoparium*	[8,31]
**46**	5-hydroxy-4′-methoxy-6-prenyl-1‴, 2‴-peroxyl-3‴,3‴-dimethyidihydro pyrano [5,6:8,7]-isoflavone	*H. scoparium*	[8,31]
**47**	3-[4-hydroxyphentl-6,7-dimetoxy-4H-1-benzopyran-4-one]	*H. sikkimense*	[32]
**48**	Isoformononetein	*H. sikkimense*	[32]
**49**	4′,6-dimethoxy-7-hydroxyisoflavone	*H. theinum*	[33]
**50**	Kanzonol K	*H. multijugum*	[30]
**51**	Formononetin 7-O-*β*-D-(6″-O-malonyl) -glucopyranoside	*H. polybotrys*	[34]
**52**	5-hydroxy-7-(2-hydroxyisopropyl)-4′- methoxy-9-prenylfurano [3,2-g]isoflavone	*H. scoparium*	[35]
**53**	5-hydroxy-4′-methoxy-9-prenylfurano [3,2-g]isoflavone	*H. scoparium*	[35]
**54**	5-hydroxy-4′-methoxy-7-prenylfurano [2,3-h]isoflavone	*H. scoparium*	[35]
**55**	8-hydroxydaidzein	*H. theinum*	[3]
*Chalcones*			
**56**	Isoliquiritigenin	*H. polybctrys*	[21,27]
**57**	Hedysarumine A	*H. gmelinii*	[36]
**58**	Hedysarumine B	*H. gmelinii*	[36]
**59**	Paratocarpin E	*H. gmelinii*	[36]
*Flavanols*			
**60**	(-)-catechin	*H. theinum*	[3]
**61**	(-)-epicatechin	*H. theinum*	[3]
		*H. kirghisorum*	[22,23]
**62**	(-)-epigallocatechin	*H. kirghisorum*	[22,23]
**63**	(-)-vestitol	*H. theinum*	[3,26]
**64**	Plumbocatechin	*H. kirghisorum*	[22,23]
*Xanthones*			
**65**	Mangiferin	*H. aericeum*	[16]
		*H. caucasium*	[16]
		*H. flavescens*	[37]
		*H. alpinum*	[37]
		*H. denticulatum*	[38]
		*H. komarovii*	[14]
		*H. sachalinense*	[15]
		*H. neglectum*	[12]
		*H. connatum*	[13]
		*H. alpinum*	[13]
**66**	Isomangiferin	*H. flavescens*	[37]
		*H. alpinum*	[37]
		*H. denticulatum*	[38]
		*H. connatum*	[13]
		*H. alpinum*	[13]
**67**	Glucomangiferin	*H. flavescens*	[39]
**68**	Glucoisomangiferin	*H. flavescens*	[39]
*Pterocarpans*			
**69**	(-)-medicarpin	*H. theinum*	[3,26]
		*H. polybotrys*	[6,20]
		*H. Semenovii*	[40]
**70**	1,7-dihydroxy-3.9-dimethoxy pterocarpene	*H. multijugum*	[28]
**71**	Demethylhedysarimpterocarpene A	*H. multijugum*	[30]
**72**	1,3-dihydroxy-9-methoxypterocarpene	*H. multijugum*	[30]
**73**	Hedysarimpterocarpene A	*H. multijugum*	[30]
**74**	7-hydroxyhedysarimpterocarpene B	*H. multijugum*	[30]
**75**	1,7-dihydroxy-3,9-dimethoxy-10-(3- methylbut-2-enyl)-pterocarpene	*H. multijugum*	[30]
**76**	1,7-dihydroxy-3,9-dimethoxy-8-(3- methylbut-2-enyl)-pterocarpene	*H. multijugum*	[30]
**77**	hedysarimpterocarpene B	*H. multijugum*	[30,41]
		*H. polybotrys*	[42]
**78**	3-hydroxy-9-methoxy pterocarpan	*H. polybotrys*	[4,27]
		*H. taipeicum*	[43]
		*H. semenovii*	[40]
		*H. gmelinii*	[44]
**79**	Hedysarimpterocarpene C	*H. multijugum*	[41]
		*H. polybotrys*	[42]
*Triterpennoids*			
**80**	Lupeol	*H. polybotrys*	[4]
		*H. scoparium*	[8]
		*H. sikkimense*	[32]
		*H. gmelinii*	[44]
**81**	12-hydroxylupeol	*H. scoparium*	[8]
**82**	Betulinic acid	*H. Polybctrys*	[45]
		*H. multijugum*	[28]
		*H. Semenovii*	[40]
**83**	Ursolic acid	*H. sikkimense*	[32]
**84**	Soyasapogenol	*H. gmelinii*	[44]
**85**	SoyasaponinIImethyl ester	*H. polybotrys*	[5,46]
		*H. polybotrys*	[18]
		*H. multijugum*	[47]
**86**	SoyasaponinI	*H. polybotrys*	[5,46]
		*H. polybotrys*	[18]
		*H. multijugum*	[47]
**87**	SoyasaponinII	*H. polybotrys*	[5,46]
		*H. polybctrys*	[18]
		*H. multijugum*	[47]
**88**	Soyasaponin Bg	*H. multijugum*	[47]
**89**	Squasapogenol	*H. gmelinii*	[44]
**90**	Dehydrosoyasaponin I	*H. polybotrys*	[5,46]
		*H. polybctrys*	[48]
**91**	Polybosaponin A	*H. polybctrys*	[48]
*Coumarins*			
**92**	Aureol	*H. multijugum*	[30,49]
**93**	Hedysarimcoumestan E	*H. multijugum*	[30,49]
**94**	Hedysarimcoumestan B	*H. multijugum*	[30,49]
**95**	Hedysarimcoumestan A	*H. multijugum*	[30,49]
**96**	Hedysarimcoumestan F	*H. multijugum*	[30,49]
**97**	Hedysarimcoumestan D	*H. multijugum*	[30,49]
**98**	Hedysarimcoumestan G	*H. multijugum*	[30,49]
**99**	Hedysarimcoumestan H	*H. multijugum*	[30,49]
**100**	Hedysarimcoumestan C	*H. multijugum*	[49]
**101**	Methylhedysarimcoumestan H	*H. multijugum*	[30]
**102**	1,3,9-trimethoxycoumestan	*H. multijugum*	[49]
**103**	3,9-dihydroxy coumestan	*H. gmelinii*	[44]
*Lignanoids*			
**104**	(+) syringaresinol	*H. polybotrys*	[27]
**105**	(+)-isolariciresinyl-9′-O-*β*-D-glucopyranoside	*H. setigerum*	[50]
*Alkaloids*			
**106**	Guanosine	*H. semenovii*	[40]
**107**	Docosanooic acid-2,3- dihydroxyproyl ester	*H. sikkimense*	[32]
**108**	Protocatechoic acid	*H. theinum*	[3]
**109**	Tryptophane	*H. polybctrys*	[18]
**110**	Sparaginic acid	*H. austrosibiricum*	[25]
**111**	Hypaphorine	*H. polybctrys*	[18]
**112**	Berberine	*H. setigerum*	[50]
*Sterols*			
**113**	Stigmasterol	*H. multijugum*	[29]
**114**	Daucosterol	*H. polybotrys*	[4,27]
		*H. semenovii*	[40]
		*H. scoparium*	[8]
		*H. austrosibiricum*	[17]
**115**	*β*-sitosterol	*H. polybotrys*	[4,27]
		*H. multijugum*	[28]
		*H. taipeicum*	[43]
		*H. scoparium*	[8]
		*H. austrosibiricum*	[17]
		*H. sikkimense*	[32]
		*H. gmelinii*	[44]
**116**	7*β*-hydroxysitosterol	*H. scoparium*	[8]
**117**	7*α*-hydroxysitosterol	*H. scoparium*	[8]
*Carbohydrates*			
**118**	Rhamnose	*H. polybctrys*	[7,51]
**119**	Arabinose	*H. polybctrys*	[7,51]
**120**	Xylose	*H. polybctrys*	[7,51]
**121**	Galactose	*H. polybctrys*	[7,51]
**122**	Glucose	*H. polybctrys*	[7,51]
**123**	Sucrose	*H. polybctrys*	[45]
		*H. taipeicum*	[43]
		*H. austrosibiricum*	[17]
		*H. sikkimense*	[32]
*Fatty compounds*			
**124**	*η*-hexacosanoic acid (Cerinic acid)	*H. polybctrys*	[4,27]
		*H. taipeicum*	[43]
**125**	*η*-tetracosanoic acid	*H. polybctrys*	[27]
		*H. multijugum*	[29]
		*H. scoparium*	[8]
		*H. taipeicum*	[43]
		*H. sikkimense*	[32]
		*H. theinum*	[26]
**126**	Trioleic glyceride	*H. polybctrys*	[27]
**127**	Glycerol monopalmitate	*H. polybctrys*	[27]
**128**	Behenic	*H. theinum*	[26]
**129**	Palmitic acid	*H. theinum*	[26]
		*H. gmelinii*	[44]
**130**	Linoleic acid	*H. theinum*	[26]
**131**	Oleic acid	*H. theinum*	[26]
**132**	Hexacosyl acetate	*H. taipeicum*	[43]
**133**	Hexadecanoic acid 2,3-dihydroxypropyl ester	*H. gmelinii*	[44]
**134**	Triacontyl alcohol	*H. multijugum*	[29]
**135**	n-tetracosanol	*H. scoparium*	[8]
*Benzofuran*			
**136**	EbenfuranII	*H. multijugum*	[30]
**137**	HedysarimbenzofuranB	*H. multijugum*	[30,52]
**138**	AndinermalC	*H. multijugum*	[30]
**139**	2-(2,4,6-trihydroxypheny)-3-formyl-4- hydroxy-5-methoxy-6 (3-methyl-but-2- enyl) benzofuran	*H. multijugum*	[30]
**140**	2-(2,6-dihydroxy-4-methoxypheny)-4- hydroxy-3-(hydroxymethyl)-5/6-metho xy-6/5-(3-methyl-but-2-enyl)benzofuran	*H. multijugum*	[30]
**141**	2-(2,6-dihydroxy-4-methoxypheny)-3- formyl-4-hydroxy-5/6-methoxy-6/5-(3-methyl-but-2-enyl)benzofuran	*H. multijugum*	[30]
**142**	2-(2-hydroxy-4,6-dimethoxyphenyl)-3-formyl-4-hydroxy-5/6-methoxy-6/5-(3-methyl-but-2-enyl)benzofuran	*H. multijugum*	[30]
**143**	Hedysarimbenzofuran A	*H. multijugum*	[30,52]
**144**	5-hydroxy-2-(2-hydroxy-4-methoxyphenyl)-6-methoxybenzofuran	*H. polybotrys*	[42]
**145**	6-hydroxy-2-(2-hydroxy-4-methoxyphenyl)-benzofuran	*H. polybotrys*	[42]
*Others*			
**146**	Raspberry ketone	*H. theinum*	[3,26]
**147**	Rhododendrol	*H. theinum*	[3,26]
**148**	Cetyl ferulate	*H. polybotrys*	[4,27]
**149**	Polybotrin	*H. polybotrys*	[53]
**150**	Hedysalignan A	*H. polybctrys*	[21]
**151**	4′-hydrox-y-trans-cinnamic acid docosyl ester	*H. multijugum*	[29]
**152**	Caffeic acid tetracosyl ester	*H. multijugum*	[29]
**153**	p-hydroxybenzoic acid	*H. setigerum*	[50]
**154**	*(E)*-3-(4-hydroxy-2-methoxyphenyl)-prope noicacid4-hydroxy-3-methoxyphenyl ester	*H. polybotrys*	[54]
**155**	Protocatechoic acid	*H. setigerum*	[50]

Among the important chemical constituents, flavonoids are usually regarded as the main groups of metabolites and quality control markers in genus *Hedysarum.* Polysaccharides with perfect pharmacological activities are important research direction in future.

### Flavonoids

Flavonoids are a large group of polyphenolic compounds found in genus *Hedysarum*. So far, 79 flavonoids (flavones, flavonones, isoflavones, chalcones, flavanols, xanthones, and pterocarpans) have been identified and categorized based on chemical structure. Flavones are the most common and predominant flavonoids in genus *Hedysarum*. To date, 21 flavones (**1–21**) and three flavonones (**22–24**) have been isolated from plants of genus *Hedysarum*[7,8,10-12,14,16,18,20]. The flavonones are naringin and naringin-type’s derivatives and are mainly distributed in *H. multijugum* and *H. polybotrys*. Isoflavones are ubiquitous secondary metabolites in genus *Hedysarum*. So far, 31 isoflavones (**25–55**) have been isolated from plants in this genus. *H. polybotrys, H. multijugum, and H. scorparium* have particularly high concentrations of isoflavones [19-21,24,28-30,32-35]. Four chalcones (**56–59**) have been isolated from *Hedysarum* species. Isoliquiritigenin (**56**) was isolated from *H. polybotrys*, while compounds (**57–59**) were obtained from *H. gmelinii*[21,36]. Five flavanols (**60–64**) have been identified in genus *Hedysarum* species. All these compounds were found in *H. theinum* and *H. kirghisorum*[22,23]. Four xanthones (**65–68**) have been identified plants of genus *Hedysarum*. Mangiferin (**65**) was found in *H. aericeum*, *H. havescen*, *H. denticulatum*, *H. komarovii*, *H. sachalinense*, *H. neglectum*, *H. connatum,* and *H. alpinum*. Isomangiferin (**66**) was obtained from *H. havescen*, *H. denticulatum*, *H. connatum,* and *H. alpinum*. Glucomangiferin (**67**) and glucoisomangiferin (**68**) were isolated from *H. flavescents*[15,37-39]. Eleven pterocarpans (**69–79**) have been isolated and identified from genus *Hedysarum*[41,42]. These structures can be classified as isoflavones.

Although most species of genus *Hedysarum* contained different kinds of flavonoids, but the types and contents of flavonoids showed differences among species. For example, content of mangiferin and sum of xanthones in the leaves of 7 species (*H. alpinum*, *H. flavescens*, *H. austrosibiricum*, *H. neglectum*, *H. theinum*, *H. gmelinii* and *H. tschuense*), growing on the forest-steppe zone of Western Siberia (Novosibirsk) and in natural populations Republic Altai and Northern Kazakhstan was studied. The greatest amount of mangiferin and the sum of xanthones was revealed in leaves of *H. alpinum* and *H. flavescens* (to 4.3 and 6.0%). *H. austrosibiricum, H. neglectum* and *H. theinum* contained almost twice less than them. *H. gmelinii* contained about 1.0%. Xanthones were absent in leaves of *H. tschuense*. Cultivated plants synthesize more xanthones, than wild-growing [55]. Five major flavonoids in 48 batches of Radix Hedysari from different origins were simultaneously evaluated by HPLC. Among the 5 major flavonoids, ononin, formononetin-7-O-*β*-D- glucopyranoside-6″-O-malonate, formononetin and medicarpin, were detected in almost all the samples and thus can be used as marker compounds to evaluate the quality of Radix Hedysari, while naringin was not detected in most samples. Further analysis of the contents of the 4 flavonoids in different samples showed that processing procedure, harvesting time and habitats were important factors affecting the flavonoid contents of Radix Hedysari [19].

### Triterpenoids

Twelve triterpenoids (80–91) were isolated from plants of genus *Hedysarum*, most of which were distributed in *H. polybotrys*[9,17,44,45,47,48]*.*

### Coumarins

Twelve coumarins have so far been isolated from this genus. Compounds **92–102** were obtained from *H. multijugum*, while 3,9-dihydroxy coumestan (**103**) was isolated from *H. gmelinii*[9,30,49].

### Lignanoids

Only 2 lignanoids have been identified from *Hedysarum* species: syringaresinol (**104**) from *H. polybotrys* and isolariciresinyl-9′-*O*-*β*-d-glucopyranoside (**105**) from *H. setigerum*[27,50].

### Nitrogen compounds

Five alkaloids (**106–110**) and 2 amino acids (**111–112**) have been isolated from the genus [25,40,50].

### Sterols

The sterols found in genus *Hedysarum* include stigmasterol (**113**), daucosterol (**114**), sitosterol (**115**), and 2 derivations of sitosterol (**116** and **117**) [28,29].

### Carbohydrates

There are 6 carbohydrates that have been isolated from *Hedysarum* species. Five of these carbohydrates were isolated from *H. polybotrys,* including rhamnose (**118**), arabinose (**119**), xylose (**120**), galactose (**121**), and glucose (**122**). Sucrose (**123**) was obtained from *H. polybotrys*, *H. taipeicum*, *H. austrosibiricum,* and *H. sikkimense*[56,57].

### Fatty compounds

Twelve fatty compounds, including seven fatty acids (**124, 125, 128–131, 134**), 3 fatty alcohols (**127, 132, 135**), and 2 others (**126, 133**) have been identified in plants of genus *Hedysarum*[26,43].

### Benzofurans and benzene derivatizations

Ten benzofurans have been found in genus *Hedysarum*. Compounds **136–143** were isolated from *H. multijugum*. Compounds **144** and **145** were obtained from *H. polybotrys*. About 10 (**146–155**) have been obtained from *Hedysarum* species [52-54].

### Polysaccharides and condensed tannins

HPS4-1A is a new neutral heteropolysaccharide from *H. polybotrys*. The absolute and relative molecular weight of HPS4-1A was 7.386×10^4^ and above 6.68×10^5^, respectively. It was consisted of L-rhamnose, L-arabinose, D-glucose and D-galactose (1:2:1:2). HPS4-1A was proved to be a neutral sugar, with 1,6- and 1,2,6-*α*-D-galactopyranosyl and 1,5- and 1,3,5-*α*-L-arabinofuranosyl residues in backbone, and 1,4- and 1,4,6-*α*-D-glucopyranosyl and 1,2- and 1,2,4-*α*-L- rhamnofuranosyl residues in branches. Arabinose mainly connected the end of backbone, and glucose and a small quantity of arabinose mainly connected the end of branches. HPS4-1A has a random coil state conformation with monodisperse mass distribution [6].

Water soluble sulfated glucan (SHG) was isolated from *H. polybotrys* using anion-exchange and gel-permeation chromatogram. Elemental analysis indicated that SHG was a sulfated polysaccharide with small amount of sulfate groups (1.47%). The molecular weight was 1.72 × 10^5^ Da. Compositional analysis revealed that SHG was composed of glucose only. SHG was composed of *α*-D-(1→4)-linked glucopyranosyl residues, with branches at C-6 consisting of non-reducing terminal approximately every eight residues. Sulfate groups may attach to the backbone at O-6, occasionally occurring per thirty-eight anhydrous glucose units [58].

The chemical characteristics of the purified condensed tannin fractions were studied by acid-catalyzed degradation with benzyl mercaptan and electrospray ionization mass spectrometry (ESI-MS). Thiolysis revealed that epigallocatechin was the major extender unit (15-75%) while gallocatechin was the major terminal unit (50-66%), thus indicating the extractable sulla condensed tannin fraction as the prodelphinidin type [59].

### Pharmacological activities

The species of genus *Hedysarum,* such as Hongqi (*Hedysarum polybotrys* Hand.-Mazz.), showed pharmacological activity in modern experiments and are potential antioxidant, anti-aging, anti-tumor, immune system regulatory, and anti-diabetic agents.

### Antioxidant activity

Free radicals are formed by various environmental chemicals and the endogenous metabolism of plants. With the development of biomedical science, the involvement of free radicals in many diseases, such as brain dysfunction, cancer, and heart disease, has become well known. Antioxidant substances that fight free radicals have a crucial role in human health. Some of the chemicals extracted from plants of genus *Hedysarum* are reported to possess strong antioxidant properties. For example, polysaccharide HPS-3 from the radix of *H. polybotrys* (Hedysari, Hongqi) showed antioxidant activity in superoxide anion (O^2-^), hydroxyl radical (·OH), 2,2-diphenyl-1-picrylhydrazyl (DPPH) free radical-scavenging, and H_2_O_2_ assays. At concentrations of 0.05 to 5.00 mg/mL, the maximum removal efficiency for O^2-^, H_2_O_2_,·OH, and DPPH were 55.92%, 59.32%, 53.69%, and 87.66%, respectively. The effects were concentration dependent [60].

Compound **27**, isolated from *H. polybotrys,* can improve the survival rate of SH-SY5Y cells and H_2_O_2_-injured DJ-1 gene-transfected SH-SY5Y cells, as indicated by the 3-(4,5-dimethylthiazol-2-yl)-2,5-diphenyl tetrazolium bromide (MTT) method. The addition of compound **27** (20 mg/L) more obviously improved the cell viability for DJ-1 gene transfected SH-SY5Y cells (cell viability of 91%) than the normal SH-SY5Y cells (cell viability of 83%). This indicates that compound **27** had a protective effect against the oxidative damage induced by hydrogen peroxide [18].

### Anti-aging activity

Aging is a natural process. Anti-aging compounds can suppress and retard the aging process of the body. A polysaccharide found in *H. polybotrys* (HPS) can prompt protein synthesis by affecting cellular RNA content, thereby repairing cell dysfunction caused by aging and other reasons [61]. HPS directly clears lipid peroxides (LPO) and significantly improve the superoxide dismutase (SOD) activity of rabbit aorta smooth muscle cells (SMC), to a greater degree than a positive control, VE. Moreover, there is a significant improvement of SOD content in the erythrocytes of aged rats, which indicates that the anti-aging activity of HPS in aged rats is due to activation of antioxidases [62]. Aqueous extracts of *H. austrosibiricum* from Xinjiang province significantly decreased the malondialdehyde (MDA) content of liver and brain tissues of aging mice after induction by d-galactose. These extracts also markedly increased the activities of SOD and glutathion peroxidase (GSH-Px). In addition, monoamine oxidase (MAO) activity in the brain tissue decreased and the spleen and thymus indexes were elevated after treatment with the aqueous extract. *H. austrosibiricum* showed anti-aging activity by eliminating free radicals and activating antioxidases [63].

### Immune system regulation activity

The immune system finds and removes foreign pathogenic microorganisms that cause fluctuations in the internal environment of the body. A polysaccharide from *H. polybotrys* (HPS) has obvious protective effects on the immune system that are similar to *Astragalus membranaceus* polysaccharide (APS). Both HPS and APS can increase the celiac macrophage phagocytic function of mice and correct inhibition of cell-mediated immunity caused by cyclophnosphamide (CY) and PDS. HPS and APS also improve lymphocyte transformation *in vivo* and significantly increase the concentration of phenol acetate esterase-positive (ANAE) lymphocytes in the fluid sampled [64]. HPS enhances humoral and cellular immune function. The lymphocyte proliferation rate *in vivo* was remarkably increased (*P* < 0.001) and inhibition of CY was completely corrected (*P* < 0.001) by HPS (0.5 g/kg) [65].

RHTS (total saponins extracted from *H. polybotrys*) obviously improved immune function suppressed by CY. The index for the function of the thymus and the spleen were increased. The phagocytic index and phagocytic percentage of intraperitoneal macrophages were significantly increased (*P* < 0. 05). CaM levels in cells of thymocytes, erythrocytes, and macrophages showed obvious correlation with immune function, which suggests that the Ca^2+^-CaM signal system plays an important role in the immune cell activation process [66]. Aqueous extracts of *H. polybotrys* and *A. membranaceus* intensify the functions of normal macrophages and those treated with 10 ng/mL LPS, as indicated by NO released by the cells. The activities of *H. polybotrys* and *A. membranaceus* ethanol extracts were not better than those of the aqueous extracts [67].

The regulating effects of Hedysari Radix and Astragali Radix alternative classic tonification prescriptions on humoral immunity in immunosuppressed mice were compared. The immunosuppressed mouse model was induced by cyclophosphamide. The mice were administered intragastically with the same dose of Hedysari Radix and Astragali Radix alternative the formula Buzhong Yiqi Yangxue, Yupingfeng oral liquid and Fuqi Zhihan granules for antagonistic experiments *in vivo*. Spleen index, HC50, CD19^+^ B lymphocyte subgroup and content of serum IL-4 were detected after treatment. Both groups of Hedyseri Radix and Astragali Radix could antagonize immunosuppressive action caused by cyclophosphamide. They both could significantly raise spleen index, HC50, CD19^+^ B lymphocyte subgroup and content of serum IL-4 in different degree. Yupingfeng water extraction of Hedysari Radix substitute Astragali Radix was better than Yupingfeng oral liquid in raising spleen index. There were no significant differences among the rest Hedysari Radix and Astragali Radix alternative groups. Hedysari Radix compatibility with other drugs compared with original prescription has similar role in humoral immunity regulation [68].

### Anti-tumor activity

Malignant tumors (cancer) are among the leading causes of death. The main bioactive chemicals found in plants of genus *Hedysarum* play an important role in the prevention of several tumor cell types. MTT assays revealed that HPS-1 that was obtained from the roots of *H. polybotrys* significantly inhibited the proliferation of human hepatocellular carcinoma HEP-G2 cells and human gastric cancer MGC-803 cells *in vitro*. At concentrations from 50 to 400 μg/mL, HPS-1 significantly inhibited the proliferation of HEP-G2 cells (*P* < 0.05) in a concentration-dependent manner. At 400 μg/mL, HPS-1 had an inhibition ratio of 40.0%. However, although HPS-1 also significantly suppressed MGC-803 cells (*P* < 0.05), there was no clear relationship between the concentration of HPS-1 and its effects. These results indicate that HPS-1 has potential for cancer therapy [69]. In addition, the cytotoxicity of the purified compounds against human cancer cell line HepG2 was evaluated using the MTT method. Compound **78** showed inhibitory activity on HepG2 with IC_50_ values of 10.69 μmol/L [27].

### Anti-diabetic properties

HPS is the principal active fraction responsible for the anti-diabetic properties of *H. polybotrys.* In ones study of the hypoglycemic activity of HPS, crude HPS was isolated, purified, and divided into 4 fractions of different molecular weight ranges. ALX treatment of mice produced a significant increase in fasting hyperglycemia, which was counteracted by treatment with HPS1, HPS3, and HPS4. A dose of 200 mg/kg HPS3 caused the maximum reduction in blood glucose level (56% at 12 days of HPS3 administration) and this effect was greater than that of the oral hypoglycemic agent metformin. Plasma insulin concentration and ISI were significantly higher in the HPS3-treated mice than in mice of the diabetes mellitus (DM) model control group. Moreover, HPS3 inhibited the secretion of tumor necrosis factor-*α* (TNF-*α*), leptin, and free fatty acid (FFA) levels by improving insulin secretion, promoting glucose uptake, suppressing gluconeogenic precursors, and decreasing glucose oxidation and output. Thus, HPS3 improved insulin resistance in diabetic mice. The beneficial effects of HPS3 also included the reduction of lipid peroxidation (increased NOS and SOD activity, increased T-AOC and NO, as well as decreased MDA) in STZ-induced diabetic rats [70].

### Effects on the peripheral nervous system

The effects of HPS on sciatic nerve regeneration in rats for 6 weeks following clamping of the nerve (HPS was administrated orally as 2 mL liquid daily, 0.25 g HPS/mL) were examined. HPS improved sciatic function index (SFI) values, tibial function index (TFI) values, peroneal nerve function index (PFI) values, conduction velocity, and the number of regenerated myelinated nerve fibers, suggesting the potential clinical application of HPS for the treatment of peripheral nerve injury in humans [71].

### Others

*H. polybotrys* has significant protective effect against heart and brain hypoxia. In study of Yasuda-chiari cerebral ischemia anoxia, HPS from *H. polybotrys* had remarkable respiration prolonged abilities (*P* <0.001) as compared to the control group. At the concentration of 0, 4, and 8 mg/g for HPS, the respiration lasted 14.3 ± 3.9 s, 22.4 ± 0.9 s, and 22.8 ± 1.3 s, respectively, representing an increase of 56.7% for 4 mg/g and 59.4% for 8 mg/g. HPS also prolonged the survival time of mice with myocardial ischemia induced by isoprenaline (ISOP) [72]. In addition, *H. polybotrys* also exhibits tonification, growth promotion, analgesic, anti-inflammatory and antiviral activity [65].

## Conclusions

In the literature, chemical investigations and pharmacological activity have been reported for only 21 of the 300 known *Hedysarum* species. However, members of *Hedysarum*, such as *H. polybotrys* and *H. austrosibiricum*, clearly possess significant pharmacological potential, especially in the treatment of immune disorders, cancer, diabetes, and hypertension. Investigation into the chemical constituents of plants of genus *Hedysarum* revealed diverse compounds, including flavonoids, triterpenes, coumarins, lignanoids, nitrogen compounds, sterols, carbohydrates, fatty compounds, benzofuran, and polysaccharides. It should be emphasized that polysaccharides from *H. polybotrys* and *H. austrosibiricum* had particularly prominent immunomodulatory, antioxidant, and anti-diabetic activity, in addition to some other biological properties. Previous studies have provided an empirical base for the medicinal use of *Hedysarum* species. It is important to note that the safety and toxicity of *Hedysarum* species have not been explored. There is no published overdose or toxicity data for these species. Therefore, the toxicities of traditional remedies and isolated chemical compounds should be further assessed.

## Competing interests

The authors declare that they have no competing interests.

## Authors’ contributions

DY, ZN, LY, LL, and ZC were involved in preparing the manuscript. LM and TD participated in discussions of views represented in the paper. All authors have read and approved the final manuscript.

## Supplementary Material

Additional file 1 **The chemical structure of compounds isolated from plants of genus *Hedysarum.*** Click here for file
