# Supplementary material for: Phytochemicals and biological studies of plants in genus Hedysarum
Source: Chem Cent J. 2013 Jul 18;7:124. doi: 10.1186/1752-153X-7-124 (PMC3727964; doi:10.1186/1752-153X-7-124)
Supplement: Additional file 1 — The chemical structure of compounds isolated from plants of genus Hedysarum. [file 1752-153X-7-124-S1.doc]

**Figure S1. The chemical structure of compounds isolated from plants of genus *Hedysarum***

**1 2**

|  | R_1_ | R_2_ | R_3_ |
| --- | --- | --- | --- |
| **3** | OH | H | H |
| **4** | OCH_3_ | H | H |
| **5** | OH | OCH_3_ | OH |
| **6** | OCH_3_ | H | H |

**16**

|  | R_1_ | R_2_ | R_3_ | R_4_ | R_5_ | R_6_ |
| --- | --- | --- | --- | --- | --- | --- |
| **7** | OH | H | H | ORha | OH | H |
| **8** | OH | H | H | O-L-Ara (*α*) | OH | H |
| **9** | OH | H | H | O-L-Ara (*β*) | OH | H |
| **10** | H | H | H | H | OH | Rha-(1→2)-Glc |
| **11** | H | CH_3_ | H | H | OH | Rha-(1→6)-Glc |
| **12** | OH | CH_3_ | H | H | OH | Rha-(1→6)-Glc |
| **13** | H | H | H | OAra | OH | H |
| **14** | OH | H | H | ORha-(1→6)-Glc | OH | H |
| **15** | H | CH_3_ | H | H | OH | α-L-Rha(1→2)-[α-L-Rha (1→6)] β-D-Glc |

| **17** | R_1_= OH | R_2_= OH |
| --- | --- | --- |
| **18** | R_1_= OH | R_2_= H |
| **19** | R_1_= OGlc | R_2_= OH |
| **20** | R_1_= OGal | R_2_= OH |
| **21** | R_1_= OGlc | R_2_= OH |

**22 23**

**24 25**

**26** R= OGlc-6"-O-malonate

**27** R= OGlc

|  | R_1_ | R_2_ | R_3_ | R_4_ | R_5_ |
| --- | --- | --- | --- | --- | --- |
| **28** | H | OH | H | H | H |
| **29** | H | OH | H | H | OH |
| **30** | H | OH | (CH_3_)_2_C=CHCH_2_ | OH | H |
| **31** | (CH_3_)_2_C=CHCH_2_ | OH | (CH_3_)_2_C=CHCH_2_ | OH | H |
| **32** | H | OH | H | OH | H |
| **33** | (CH_3_)_2_C=CHCH_2_ | OH | H | OH | H |
| **34** | H | OH | OCH_3_ | H | H |
| **35** | H | OGlc | H | OH | H |
| **36** | H | OGlc | OCH_3_ | H | H |
| **37** | H | OGlc | H | H | OH |

**38** R= H **40**

**39** R= OH

**41 42**

**43**  **44**

**45 46**

|  | R_1_ | R_2_ | R_3_ | R_4_ | R_5_ | R_6_ |
| --- | --- | --- | --- | --- | --- | --- |
| **47** | OCH_3_ | OCH_3_ | H | H | H | OH |
| **48** | OCH_3_ | H | H | H | H | OH |
| **49** | OH | OCH_3_ | H | H | H | OCH_3_ |
| **50** | OCH_3_ | (CH_3_)_2_C=CHCH_2_ | OH | OH | (CH_3_)_2_C=CHCH_2_ | OH |

**51**

**52**

**53 54**

1. **56**

**57 58**

**59 60**

**61** R= H  **63**

**62** R= OH

**64 69**

|  | R_1_ | R_2_ | R_3_ |
| --- | --- | --- | --- |
| **65** | OH | OAra | OH |
| **66** | OGlc | OH | H |
| **67** | H | OH | OGlc |
| **68** | OGlc | OH | H |

**79**

|  | R_1_ | R_2_ | R_3_ | R_4_ | R_5_ | R_6_ |
| --- | --- | --- | --- | --- | --- | --- |
| **70** | OH | OCH_3_ | OH | H | OCH_3_ | H |
| **71** | OH | OH | H | OCH_3_ | H | OH |
| **72** | OH | OH | H | OCH_3_ | H | H |
| **73** | OCH_3_ | OH | H | OCH_3_ | H | OH |
| **74** | OH | OH | (CH_3_)_2_CHCH_2_ | OCH_3_ | H | OH |
| **75** | OCH_3_ | OH | (CH_3_)_2_CHCH_2_ | OCH_3_ | H | OH |
| **76** | OCH_3_ | OH | H | OCH_3_ | (CH_3_)_2_CHCH_2_ | OH |
| **77** | OH | OH | (CH_3_)_2_CHCH_2_ | OCH_3_ | H | H |
| **78** | OH | H | H | OCH_3_ | H | H |

**80** R= H **82**

**81** R= OH

|  | R_1_ | R_2_ | R_3_ | R_4_ | R_5_ | **88** R_1_ = GluA-Ara-Rha |
| --- | --- | --- | --- | --- | --- | --- |
| **83** | CH_3_ | CH_3_ | H | COOH | CH_3_ |  |
| **84** | H | (CH_3_)_2_ | O= | CH_3_ | CH_2_OH |  |

**85** R_1_ = GlcA-Ara-Rha, R_2_= β-OH **89**

**86** R_1_ = GluA-Gal-Rha, R_2_= β-OH

**87** R_1_ = GluA-Ara-Rha, R_2_= β-OH

**90**

**91**

|  | R_1_ | R_2_ | R_3_ | R_4_ | R_5_ | R_6_ |
| --- | --- | --- | --- | --- | --- | --- |
| **92** | OH | OH | H | OH | H | H |
| **93** | OH | OH | H | OCH_3_ | OCH_3_ | H |
| **94** | OH | OH | H | OCH_3_ | H | H |
| **95** | OCH_3_ | OH | H | OCH_3_ | H | H |
| **96** | OCH_3_ | OH | H | OCH_3_ | H | OH |
| **97** | OH | OH | (CH_3_)_2_CHCH_2_ | OCH_3_ | H | H |
| **98** | OH | OH | (CH_3_)_2_CHCH_2_ | OCH_3_ | H | OH |
| **99** | OH | OH | H | (CH_3_)_2_CHCH_2_ | OCH_3_ | OH |
| **100** | OH | OGlc | H | OH | H | H |
| **101** | OCH_3_ | OH | H | OCH_3_ | (CH_3_)_2_CHCH_2_ | OH |
| **102** | OCH_3_ | OCH_3_ | H | OCH_3_ | H | H |
| **103** | OH | H | H | OH | H | H |

**104 105**

**106 107**

**108**   **109**   **110**

**111 112**

|  | R_1_ | R_2_ | R_3_ |  | R |
| --- | --- | --- | --- | --- | --- |
| **113** | OH | H | CH_3_ | **116** | α-OH |
| **114** | OGlc | CH_3_ | H | **117** | β-OH |
| **115** | OH | CH_3_ | H |  |  |

**118 119 120 121**

**122 123 124 125**

**126**

**127 128**  **129**

**130**

**131**

**132 133**

CH_3_(CH_2_)_21_OH

**134 135**

|  | R_1_ | R_2_ | R_3_ | R_4_ | R_5_ | R_6_ | R_7_ | R_8_ |
| --- | --- | --- | --- | --- | --- | --- | --- | --- |
| **136** | CHO | H | OH | CHO | H | OH | H | OH |
| **137** | CHO | H | OH | CHO | OH | OCH_3_ | H | OH |
| **138** | CHO | H | OH | CHO | H | OH | OH | OCH_3_ |
| **139** | (CH_3_)_2_C=CHCH_2_ | OCH_3_ | OH | CHO | OH | OH | H | OH |
| **140** | (CH_3_)_2_C=CHCH_2_ | OCH_3_ | OH | CH_2_OH | OH | OCH_3_ | H | OH |
| **141** | (CH_3_)_2_C=CHCH_2_ | OCH_3_ | OH | CHO | OH | OCH_3_ | H | OH |
| **142** | (CH_3_)_2_C=CHCH_2_ | OCH_3_ | OH | CHO | OCH_3_ | OCH_3_ | H | OH |
| **143** | OCH_3_ | (CH_3_)_2_C=CHCH_2_ | OH | CHO | OH | OH | H | OH |

**144 145**

**146 147 148**

1. **153** R_1_= OH, R_2_= H

**155** R_1_= H, R_2_= OH

**150 151**

**152**  **154**
